# Health costs in anthroposophic therapy users: a two-year prospective cohort study

**DOI:** 10.1186/1472-6963-6-65

**Published:** 2006-06-02

**Authors:** Harald J Hamre, Claudia M Witt, Anja Glockmann, Renatus Ziegler, Stefan N Willich, Helmut Kiene

**Affiliations:** 1 Institute for Applied Epistemology and Medical Methodology, Böcklerstr. 5, 79110 Freiburg, Germany; 2 Institute of Social Medicine, Epidemiology, and Health Economics, Charité University Medical Center, Campus Mitte, 10098 Berlin, Germany; 3 Society for Cancer Research, Kirschweg 9, 4144 Arlesheim, Switzerland

## Abstract

**Background:**

Anthroposophic therapies (counselling, special medication, art, eurythmy movement, and rhythmical massage) aim to stimulate long-term self-healing processes, which theoretically could lead to a reduction of healthcare use. In a prospective two-year cohort study, anthroposophic therapies were followed by a reduction of chronic disease symptoms and improvement of quality of life. The purpose of this analysis was to describe health costs in users of anthroposophic therapies.

**Methods:**

717 consecutive outpatients from 134 medical practices in Germany, starting anthroposophic therapies for chronic diseases, participated in a prospective cohort study. We analysed direct health costs (anthroposophic therapies, physician and dentist consultations, psychotherapy, medication, physiotherapy, ergotherapy, hospital treatment, rehabilitation) and indirect costs (sick leave compensation) in the pre-study year and the first two study years. Costs were calculated from resource utilisation, documented by patient self-reporting. Data were collected from January 1999 to April 2003.

**Results:**

Total health costs in the first study year (bootstrap mean 3,297 Euro; 95% confidence interval 95%-CI 3,157 Euro to 3,923 Euro) did not differ significantly from the pre-study year (3,186 Euro; 95%-CI 3,037 Euro to 3,711 Euro), whereas in the second year, costs (2,771 Euro; 95%-CI 2,647 Euro to 3,256 Euro) were significantly reduced by 416 Euro (95%-CI 264 Euro to 960 Euro) compared to the pre-study year. In each period hospitalisation and sick-leave together amounted to more than half of the total health costs. Anthroposophic therapies and medication amounted to 3%, 15%, and 8% of total health costs in the pre-study year, first year, and second study year, respectively. The cost reduction in the second year was largely accounted for by a decrease of inpatient hospitalisation, leading to a hospital cost reduction of 519 Euro (95%-CI 377 Euro to 904 Euro) compared to the pre-study year.

**Conclusion:**

In patients starting anthroposophic therapies for chronic disease, total health costs did not increase in the first year, and were reduced in the second year. This reduction was largely explained by a decrease of inpatient hospitalisation. Within the limits of a pre-post design, study findings suggest that anthroposophic therapies are not associated with a relevant increase in total health costs.

## Background

Complementary therapies are popular and extensively used. In Germany and Switzerland some complementary therapies are reimbursed within health care budgets. In these countries there has been a debate as to whether reimbursement of complementary therapies may lead to increased overall health expenditures [1].

Anthroposophic medicine (AM) was founded in the 1920s by Rudolf Steiner and Ita Wegman [2]. AM therapies are provided by physicians (counselling, AM medication) and non-medical therapists (AM art, eurythmy movement, and massage therapy) in inpatient and outpatient settings. AM aims to stimulate long-term self-healing processes in patients [3], which theoretically could lead to a reduction of healthcare use. Observational data suggest AM can be associated with cost savings [4], but no studies of total health costs have been undertaken.

The Anthroposophic Medicine Outcomes Study (AMOS) [5] provided an opportunity to investigate total health costs in AM users. AMOS is a prospective cohort study of outpatients starting AM therapies for chronic disease. The study was initiated by a health insurance company in conjunction with a health benefit project including reimbursement of AM therapies [5]. AM therapies were implemented during the first 3–6 months after study enrolment and were followed by a substantial reduction of disease severity and an improvement of quality of life [5]. In a first cost analysis, the pre-study year was compared to the first study year [5]. Here we present a cost analysis with a larger patient sample, including costs in the second study year.

## Methods

### Objective and design

The objective was to study health costs in AM therapy users from the societal perspective. For this purpose, we analysed health service use in a prospective cohort study of patients starting AM therapies for chronic disease, and calculated health costs. We calculated total (direct and indirect) costs in the first and second years after study entry and compared them to costs in the pre-study year. For each year we determined the relative size of AM therapy costs vs. total health costs. Exploratory subgroup analyses were performed for different age and therapy groups.

### Setting, participants, and therapy

Participating physicians were certified by the Physicians' Association for Anthroposophical Medicine in Germany and had an office-based practice or worked in outpatient clinics in Germany. The physicians recruited consecutive patients starting AM therapy. Patients enrolled in the period 1 Jan 1999 to 31 March 2001 were included in the present analysis (18- and 24-month follow-ups were not performed for patients enrolled before 1 Jan 1999; n = 87) if they fulfilled eligibility criteria:

Inclusion criteria: (a) Outpatients aged 1–75 years, (b) referral to AM therapy (art, eurythmy or rhythmical massage), or initial AM-related consultation ≥ 30 min for any indication (main diagnosis), (c) at least three out of five follow-up questionnaires returned within the first two study years.

Exclusion criteria: previous AM therapy (art/eurythmy/rhythmical massage/AM-related consultation ≥ 30 min) for main diagnosis, respectively.

Therapy: Patients were treated according to the physician's discretion.

### Outcome measures

Health costs, regardless of diagnosis, in the pre-study year and in the first and second study years: direct health costs (AM therapies, physician and dentist consultations, psychotherapy, medication, physiotherapy, ergotherapy, inpatient hospital and rehabilitation treatment), indirect costs (sick leave compensation).

### Data collection

All data were documented with questionnaires sent in sealed envelopes to the study office. Physicians documented eligibility criteria and baseline health status; all other items were documented by patients. Patient responses were not made available to physicians. Physicians were compensated 40 Euro per included and fully documented patient, while patients received no compensation.

Data were entered twice by two different persons into Microsoft^® ^Access 97. The two datasets were compared and discrepancies resolved by checking with the original data.

### Quality assurance, adherence to regulations

The study was approved by the Ethics Committee of the Faculty of Medicine Charité, Humboldt University Berlin, and was conducted according to the Helsinki Declaration and ICH-GCP guidelines. Written informed consent was obtained from all patients before enrolment.

### Data analysis

Data analysis (SPSS^® ^13.0.1, StatXact^® ^5.0.3, S-PLUS^® ^7.0) was performed on all patients fulfilling eligibility criteria. For total and hospital costs, bootstrap means with bias-corrected and accelerated (BCa) bootstrap 95% confidence intervals (95%-CI) were calculated, using 2000 replications per analysis [6]. For other continuous data Wilcoxon Signed-Rank test was used for paired samples, Mann-Whitney U-test for independent samples; median differences with 95%-CI were estimated according to Hodges and Lehmann [7]. For binominal data McNemar test and Fisher's exact test were used. All tests were two-tailed. Significance criteria were p < 0.05 and 95%-CI not including 0.

Resource utilisation (therapies and health services) were analysed replacing missing data for each item and follow-up period by the group mean value. Costs were analysed from the perspective of the payer (employer: sick-leave costs for first six weeks; statutory health insurance: direct costs and sick-leave costs beyond first six weeks). Patient co-payment was not subtracted from direct costs.

Unit costs (Table 1) were calculated from average costs per item in Germany, year 2000 value (physicians' and dentists' fees, medication, hospital, rehabilitation, sick-leave costs [8,9]) or from reimbursement fees regulated in health care benefit catalogues (AM therapies, paraclinical investigations, psychotherapy, physiotherapy, ergotherapy [10-13]).

Hospital costs were calculated from average costs in each German federal state [14]. Physicians' fees were calculated from average fees of general practitioners + 12 specialist categories in the Accounting Data Record Panel of the Central Research Institute of Ambulatory Health Care in Germany [15]. Costs for paraclinical investigations (x-rays, computer tomography scans, nuclear magnetic resonance imaging and scintigrams) were calculated separately [11]. Costs of AM medication (any medication produced by the pharmaceutical companies Abnoba Arzneimittel GmbH, Helixor Heilmittel GmbH & Co, WALA Heilmittel GmbH, and Weleda AG) were calculated from average costs in 51 different price groups. Costs of other medications were calculated from national average costs in 86 Anatomical Therapeutic Chemical subgroups [16]. Sick-leave costs were calculated from national average gender-specific earnings for civil servants, salaried employees, and wage earners (100% compensation for sick-leave days 1–42, 70% compensation thereafter) [9]. Costs were not discounted.

## Results

### Participating physicians

153 physicians screened patients. 134 physicians had evaluable patients; these physicians did not differ significantly from all AM-certified physicians in Germany (n = 362) regarding gender (56.7% vs. 62.2% males), age (mean 46.3 ± 7.2 vs. 47.5 ± 7.9 years), number of years in practice (18.5 ± 7.5 vs. 18.9 ± 7.3 years), or the proportion of primary care physicians (86.6% vs. 85.0%).

### Patient recruitment and follow-up

**Figure 1:**
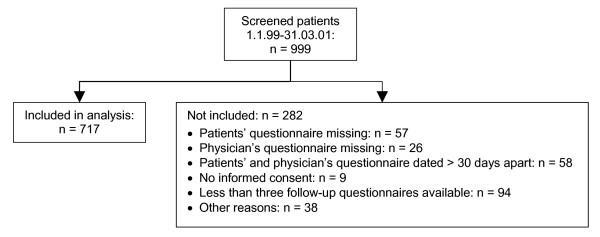
Patient recruitment and follow-up.

From 1 Jan 1999 to 31 March 2001, a total of 999 patients were screened for inclusion. 717 patients fulfilled all eligibility criteria and were included in the analysis (Figure 1). Included and not included patients did not differ significantly regarding age, gender, diagnosis, disease duration, or baseline symptom severity. The last patient follow-up ensued on 30 April 2003. 74.3% (533/717) of patients were enrolled by general practitioners, 10.2% by internists, 5.7% by paediatricians, and 9.8% by other specialist physicians. Physicians' setting was primary care practice (87.2% of patients, n = 625/717), referral practice (7.9%), and outpatient clinic (4.9%). Each physician enrolled median 3.0 patients (interquartile range IQR 2.0–7.0 patients).

### Baseline characteristics

Most frequent main diagnoses, classified by ICD-10 (International Classification of Diseases, Tenth Edition), were F00-F99 Mental Disorders (31.8%, 228/717 patients), M00-M99 Musculoskeletal Diseases (19.0%), J00-J99 Respiratory Diseases (8.8%), and G00-G99 Nervous System Diseases (7.0%). Median disease duration was 3.0 (IQR 0.9–8.0) years. 78.8% (565/717) of patients had at least one comorbid disease, median 1.0 (IQR 1.0–3.0) comorbid diseases per patient. Most common comorbid diseases, classified by ICD-10, were M00-M99 Musculoskeletal Diseases (15.3%, 184/1206 diagnoses), F00-F99 Mental Disorders (14.3%), I00-I99 Circulatory System Diseases (8.3%), E00-E90 Endocrine, Nutritional and Metabolic Diseases, and J00-J99 Respiratory Diseases (8.0%). Patients were recruited from 15 of 16 German federal states. Median age was 39.0 (IQR 22.0–48.0) years. Compared to the German population, patients had higher educational and occupational levels, had less daily alcohol consumers and regular smokers, and were less overweight; patients' socio-demographic status was similar to the population regarding low-income, living alone, severe disability status, and sport; and less favourable for work disability pension and sick-leave (Table 2).

### Resource utilisation

Compared to the pre-study year, the use of AM therapies was increased during both the first and the second study year, medication use and psychotherapy increased in the first year but not in the second year, whereas the number of hospital and rehabilitation days decreased progressively and were significantly decreased in the second year (Table 3).

### Costs

Total health costs averaged 3,186 Euro (bootstrap mean 3,186 Euro; 95%-CI 3,037 Euro to 3,711 Euro) per patient in the pre-study year and 3,302 Euro (bootstrap mean 3,297 Euro; 95%-CI 3,157 Euro to 3,923 Euro) in the first study year, an increase of 123 Euro (95%-CI -391 Euro to +320 Euro) from the pre-study year. In the second study year, total costs were 2,768 Euro (bootstrap mean 2,771 Euro; 95%-CI 2,647 Euro to 3,256 Euro) a decrease of 416 Euro (95%-CI 264 Euro to 960 Euro) from the pre-study year (Table 4). Cost distribution was highly skewed in all periods; in the first study year, 5% of patients caused 38% of all costs. In each year hospital costs and sick-leave compensation together amounted to more than half of the costs. Costs of AM therapies and medication amounted to 3%, 15%, and 8% of total health costs in the pre-study year, first year, and second study year, respectively.

In the first study year the largest cost differences from the pre-study year were observed for AM therapies (nominal increase of 347 Euro per patient) and inpatient hospital costs (nominal decrease of 310 Euro, estimated decrease of 314 Euro, 95%-CI 130 Euro to 753 Euro); in the second year the largest differences from the pre-study year were again for AM therapies (nominal increase of 108 Euro) and hospital costs (nominal decrease of 513 Euro, estimated decrease of 519 Euro; 95%-CI 377 Euro to 904 Euro). Other costs differed little (differences < 50 Euro per patient and year).

Total health costs were analysed in age and therapy groups (Table 5). Average costs in the first study year varied by a factor of 3.3 between age groups (1–19 years: 1416 Euro, 40–59 years: 4646 Euro) and by a factor of 1.5 between AM therapy groups (medical: 2614 Euro, art therapy: 3706 Euro).

## Discussion

We analysed direct and indirect health costs in German outpatients starting AM therapies for chronic disease under routine conditions. Compared to the pre-study year, costs did not differ significantly in the first year after enrolment, whereas in the second year costs were significantly reduced by 13% (416 Euro per patient).

Strengths of this study include a large patient sample, a long follow-up period, high follow-up rates, and the participation of 37% of all AM-certified physicians in Germany. Participants resembled all eligible physicians with respect to socio-demographic characteristics, and included patients resembled not included, screened patients regarding baseline characteristics. These features suggest that the study to a high degree mirrors contemporary AM use in outpatient settings. Moreover, since patients with all diagnoses were included, our study offers a comprehensive picture of AM practice. Therefore, in the present early phase of economic AM evaluation, the inclusion of all diagnoses is an advantage. On the other hand, we did not attempt to separate disease-specific costs from overall health costs. Our analysis is comprehensive, including cost domains (physician and dentist services, psychotherapy, physiotherapy, ergotherapy, medication, inpatient treatment, sick-leave compensation) amounting to 87% of healthcare expenditures of the German Statutory Health System [8] (13% not analysed: dentures, medical appliances, nursing, patient transport, and health prevention programs).

A limitation of the study is the absence of a comparison group. We do not know if in similar patients in similar settings receiving conventional or no treatment, costs would have increased, been stable, or been reduced.

Another limitation is that cost analysis was not based on direct cost measurement but on patient self-reporting of resource utilisation, which can be affected by recall bias. In this pre-post analysis, however, any systematic recall bias would probably have been conservative, making results appear less favourable. The reason is: While at study entry patients were asked about therapies and health services during the preceding 12 months, these items were thereafter asked every six months (medicine use also after three months). Since patients' recall of resource utilisation declines over time with a net tendency towards under-reporting [17], under-reporting is more likely for the 12-month pre-study period than for the shorter periods after study entry.

Dropout is unlikely to have biased the analysis of resource utilisation: For this analysis, 88% of patients were evaluable. Moreover, there is no a priori reason to assume that therapies and health services are used differently by dropouts and respondents.

Since patients were treated by AM physicians who could possibly have an interest in AM therapies having favourable outcomes, study data were largely collected by patients and not physicians. For this analysis, any bias affecting physician's documentation would not affect outcomes (resource utilisation), since these outcomes were documented by patients. Also, physicians' documentation of baseline health status (main and comorbid diagnoses) did not affect patient recruitment, since patients were enrolled regardless of diagnoses.

Major determinants of cost changes were an increased use of AM therapies (corresponding to a cost increase from the pre-study year of 377 Euro and 116 Euro per patient in the first and second year, respectively) and a reduction of hospitalisation (corresponding to a cost reduction of 310 Euro and 513 Euro, respectively), whereas other costs differed by less than 50 Euro per year. The increase of AM therapies is a consequence of the study inclusion criterion of patients starting new AM therapies. The reduction in hospitalisation was paralleled by a reduction of disease severity and improvement in quality of life [5] and may thus be related to successful therapies or spontaneous improvement. Another possible cause is frequent or long hospitalisation early in the course of disease (diagnosis, therapy initiation) followed by a normalisation of hospitalisation rates. Sensitivity analysis, however, suggests that this factor could at maximum explain 37% of the hospitalisation reduction in the second year (primary analysis: decrease by average 1.78 days = 100%, compared to the pre-study year; patients with disease duration of at least one year: decrease by 1.13 days = 63%).

Moreover, changes in health-care implementation may affect the frequency and duration of hospital treatment. However, during this study, the average number of hospital days per person-year in Germany decreased by only 0.21 days (1999–2003: 2.07→1.86 days) [18]. This reduction of 0.11 days per two years corresponds to only 6% of the observed reduction of 1.78 days per two years in our study patients. Therefore, the reduced hospitalisation in our study cannot be explained by changes in health-care implementation. A possible setting-related cause of reduced hospitalisation is the policy of AM general practitioners to provide more comprehensive patient care and avoid unnecessary referrals to secondary care [3,19]. Study implications: The reduction of hospital treatment in this cohort following AM therapies is in accordance with other findings: In two Dutch studies [20,21] and a British NHS audit [22] patients of AM physicians had 10%–35% less hospital days than local or national averages.

In Germany, patients may use specialist health services without referral from a primary care physician, generating additional costs. Our study is the first economic analysis of AM therapies taking into account such direct health costs generated outside the AM setting, as well as indirect costs (sick-leave compensation). In the first study year, costs of AM therapies amounted to 15% of total health costs and were largely outweighed by the reduced hospital costs; therefore, total costs were not significantly increased from the pre-study-year (as found in our previous analysis of a smaller patient sample of this study [5]), and in the second year a cost decrease of 416 Euro per patient (bootstrap 95%-CI indicating a decrease of at least 264 Euro) was found.

## Conclusion

In patients starting anthroposophic therapies for chronic disease, costs did not increase in the first year, in spite of the intensified therapy. In the second year, a reduction of costs was observed. This reduction was largely explained by a decrease of inpatient hospitalisation. Within the limits of a pre-post design, our findings suggest that anthroposophic healthcare in Germany is not associated with a relevant increase in total health costs.

## Abbreviations

AM: anthroposophic medicine, AMOS: Anthroposophic Medicine Outcomes Study

## Competing interests

HJH has received funding from WALA Heilmittel GmbH and Weleda AG, who produce anthroposophic medications. These companies did not finance this manuscript and had no influence on design, planning, conduct, analysis, interpretation, or publication of this study. Otherwise all authors declare that they have no competing interests.

## Authors' contributions

HJH, CMW, SNW, and HK contributed to study design. HJH, AG, and HK contributed to data collection. HJH, RZ, and HK wrote analysis plan, HJH, AG, and RZ analysed data. HJH was principal author of the paper, had full access to all data, and is guarantor. All authors contributed to manuscript drafting and revision and approved the final manuscript.

## Pre-publication history

The pre-publication history for this paper can be accessed here:



## Figures and Tables

**Table 1:** Cost calculation

**Health service category**	**Measurement unit**	**Categories/subgroups analysed separately**	**Unit cost (range) in Euro**	Cost type*	**Source**
AM therapies	Therapy session	Eurythmy, art, rhythmical massage	19.9 – 32.2	Fee	[10]
AM medication	Daily dose	51 price groups	0.1 – 5.2	Price	Manufacturer's price lists
Physician visit	Visit	12 specialist categories	12.3 – 28.6	Cost	[15]
Dentist visit	Visit	(excluding dental prosthesis)	62.4	Cost	[8]
Psychotherapy	Therapy session		56.1	Fee	[11]
Paraclinical investigation	Investigation	X-rays, computer tomography scans, nuclear magnetic resonance imaging, scintigrams	17.4 – 200.3	Fee	[11]
Non-AM medication	Daily dose	86 ATC groups	0.1 – 37.1	Price	[16]
Physiotherapy	Therapy session	Remedial gymnastics, exercise therapy, massage, fango packs, aerothermotherapy, other	5.7 – 13.7	Fee	[12]
Ergotherapy	Therapy session		24.9	Fee	[13]
Inpatient hospitalisation	Day	16 Federal states	271.0 – 411.0	Cost	[14]
Inpatient rehabilitation	Day		58.0	Cost	IKK Hamburg
Sick leave	Day	Days 1–42 vs. Days >42/self- employed, civil servants, salaried employees, wage earners, unemployed/males vs. females	41.5 – 113.1	Cost	[9]

Fees: Fee regulated in health benefit catalogues. Cost: Average annual costs in Germany. Price: Average pharmacy retail price in each medication subgroup.

**Table 2:** Socio-demographic data

**Items**		**Study patients**		**German primary care patients**	
		**N**	**%**	**%**	**Source**

Female gender		520/717	73%	53%	[23]
Age groups	0–19 years	176/717	25%	14%	[23]
	20–39 years	201/717	28%	27%	
	40–59 years	269/717	38%	27%	
	60–75 years	71/717	10%	21%	

		**Adult study patients**		**German population**	

"Fachhochschule" or university entrance qualification		302/545	55%	19%	[9]
University degree		132/544	24%	6%	[9]
Occupational status in the last 12 months	Gainfully employed patients				[9]
-Self-employed		47/281	17%	10%	
-Civil servants ("Beamter")		19/281	7%	7%	
-Salaried employee ("Angestellte")		189/281	67%	49%	
-Wage earners ("Arbeiter")		11/281	4%	34%	
-Other		15/281	5%	1%	
Unemployed in the last 12 months	Economically active patients	10/291	3%	10%	[9]
Living alone		106/540	20%	21%	[9]
Net family income < 900 Euro per month		62/451	14%	16%	[9]
Alcohol use daily (patients) vs. almost daily (Germany)	Male	3/102	3%	28%	[24]
	Female	11/443	2%	11%	
Regular smoking	Male	10/102	10%	37%	[25]
	Female	46/442	10%	28%	
Sports activity ≥ 1 hour weekly	Age 25–69	236/505	47%	39%	[26]
Body mass index ≥ 25 (overweight)	Male	25/101	25%	56%	[9]
	Female	111/437	25%	39%	
Permanent work disability pension		43/545	8%	3%	[27]
Severe disability status		54/545	10%	12%	[28]
Sick leave days in the last 12 months (mean ± SD)	Economically active patients	27.9 ± 58.0 days		17.0 days	[29]

**Table 3:** Health resource utilisation per patient and year

**Item**	**Pre-study year**		**0–12 months**				**12–24 months**			
	**Mean**	**± SD**	**Mean**	**± SD**	**Median difference (95%-CI) from pre-study year**	**P-value**	**Mean**	**± SD**	**Median difference (95%-CI) from pre-study year**	**P-value**

AM therapy sessions	1.58	± 5.84	12.66	± 12.70	+14.49 (13.50 to 15.00)	< 0.001	5.10	± 10.72	+6.59 (5.39 to 7.50)	< 0.001
AM medicines per day	0.44	± 0.80	0.73	± 0.88	+0.28 (0.23 to 0.34)	< 0.001	0.43	± 0.74	+0.01 (-0.03 to +0.05)	0.647
Non-AM medicines per day	0.69	± 0.99	0.77	± 1.03	+0.05 (0.01 to 0.09)	0.015	0.66	± 0.96	-0.03 (-0.07 to +0.02)	0.203
Physician and dentist visits	18.30	± 19.93	18.41	± 16.45	+0.69 (0.00 to +0.50)	0.047	17.45	± 36.42	-1.45 (-2.00 to -0.50)	0.003
Paraclinical investigations	1.08	± 1.65	0.92	± 2.03	-0.50 (-0.50 to 0.00)	< 0.001	0.83	± 1.57	-0.30 (-0.58 to -0.27)	< 0.001
Psychotherapy sessions	3.19	± 12.96	3.76	± 11.02	+2.00 (0.43 to 4.00)	0.027	3.75	± 10.69	+2.34 (1.67 to 2.86)	< 0.001
Hospital days	4.07	± 15.65	2.92	± 15.76	-2.00 (-4.24 to 0.00)	0.035	2.29	± 11.84	-1.55 (-3.00 to -0.05)	0.037
Rehabilitation days	1.97	± 8.06	1.79	± 7.31	-0.50 (-3.50 to +7.00)	0.598	1.26	± 5.54	-0.62 (-0.69 to 0.56)	0.004
Physiotherapy sessions	9.09	± 20.40	10.65	± 26.18	+1.00 (-0.55 to +4.00)	0.232	10.93	± 26.08	+1.91 (-0.20 to +4.00)	0.092
Sick leave days*	27.92	± 57.96	28.43	± 69.02	-2.07 (-6.00 to +1.50	0.209	28.44	± 64.85	-0.50 (-4.50 to +3.00)	0.614

*economically active patients, n = 291.

**Table 4:** Health costs per patient and year (Euro)

**Item**	**Pre-study year**			**0–12 months**			**12–24 months**		
	**Mean**	**± SD**	**Percentage of costs**	**Mean**	**± SD**	**Percentage of costs**	**Mean**	**± SD**	**Percentage of costs**

AM therapies	51	± 186	1.6%	398	± 400	12.1%	159	± 334	5.7%
AM medication	58	± 145	1.8%	88	± 164	2.7%	65	± 166	2.3%
**Total AM costs**	**108**	**± 244**	**3.4%**	**485**	**± 451**	**14.7%**	**224**	**± 378**	**8.1%**
Physician visits, paraclinical investigations	247	± 264	7.7%	234	± 226	7.1%	225	± 686	8.1%
Psychotherapy	179	± 727	5.6%	211	± 619	6.4%	211	± 600	7.6%
Dentist visits	155	± 221	4.9%	155	± 206	4.7%	163	± 182	5.9%
Non-AM medication	219	± 464	6.9%	259	± 507	7.8%	229	± 517	8.3%
Physiotherapy, ergotherapy	103	± 232	3.2%	112	± 286	3.4%	116	± 271	4.2%
Inpatient hospital treatment	1224	± 4646	38.4%	914	± 5052	27.7%	711	± 3741	25.7%
Inpatient rehabilitation	114	± 468	3.6%	104	± 424	3.1%	73	± 321	2.6%
**Total non-AM direct costs**	**2241**	**± 5092**	**70.3%**	**1989**	**± 5444**	**60.2%**	**1728**	**± 4160**	**62.4%**
**Total direct costs**	**2349**	**± 5158**	**73.7%**	**2474**	**± 5605**	**74.9%**	**1952**	**± 4331**	**70.5%**
Sick leave compensation	837	± 2702	26.3%	828	± 3099	25.1%	816	± 2835	29.5%
**Total costs**	**3186**	**± 6447**	**100.0%**	**3302**	**± 6798**	**100.0%**	**2768**	**± 5483**	**100.0%**

**Table 5:** Total health costs per patient and year in subgroups (Euro)

		**Pre-study year**		**0–12 months**		**12–24 months**	
	**N**	**Mean**	**± SD**	**Mean**	**± SD**	**Mean**	**± SD**

**Age groups**							
0–19 years	176	1372	± 5177	1416	± 3121	967	± 1056
20–39 years	201	3296	± 5889	3109	± 4575	3272	± 7356
40–59 years	269	4254	± 7390	4646	± 9607	3337	± 5225
60–75 years	71	3323	± 6101	3433	± 4384	3651	± 5884
**AM therapy groups**							
Eurythmy	339	3079	± 6563	3413	± 6101	2723	± 4786
Art therapy	136	3898	± 7417	3706	± 10147	2599	± 3660
Rhythmical massage	60	2601	± 3236	3845	± 6346	3300	± 4197
Medical	182	3046	± 6244	2614	± 4762	2804	± 7764
**All patients**	717	3186	± 6447	3302	6798	2768	± 5483
